# Dietary Quercetin and Kaempferol: Bioavailability and Potential Cardiovascular-Related Bioactivity in Humans

**DOI:** 10.3390/nu11102288

**Published:** 2019-09-25

**Authors:** Wijdan M. Dabeek, Melissa Ventura Marra

**Affiliations:** Division of Animal and Nutritional Sciences, West Virginia University, Morgantown, WV 26506, USA; wmdabeek@mix.wvu.edu

**Keywords:** quercetin, kaempferol, flavonols, hypertension, cardiovascular disease

## Abstract

Fruit and vegetable intake has been associated with a reduced risk of cardiovascular disease. Quercetin and kaempferol are among the most ubiquitous polyphenols in fruit and vegetables. Most of the quercetin and kaempferol in plants is attached to sugar moieties rather than in the free form. The types and attachments of sugars impact bioavailability, and thus bioactivity. This article aims to review the current literature on the bioavailability of quercetin and kaempferol from food sources and evaluate the potential cardiovascular effects in humans. Foods with the highest concentrations of quercetin and kaempferol in plants are not necessarily the most bioavailable sources. Glucoside conjugates which are found in onions appear to have the highest bioavailability in humans. The absorbed quercetin and kaempferol are rapidly metabolized in the liver and circulate as methyl, glucuronide, and sulfate metabolites. These metabolites can be measured in the blood and urine to assess bioactivity in human trials. The optimal effective dose of quercetin reported to have beneficial effect of lowering blood pressure and inflammation is 500 mg of the aglycone form. Few clinical studies have examined the potential cardiovascular effects of high intakes of quercetin- and kaempferol-rich plants. However, it is possible that a lower dosage from plant sources could be effective due to of its higher bioavailability compared to the aglycone form. Studies are needed to evaluate the potential cardiovascular benefits of plants rich in quercetin and kaempferol glycoside conjugates.

## 1. Introduction

Cardiovascular disease (CVD) remains the leading cause of death worldwide [1]. Fruit and vegetable intake has been associated with reduced risk of CVD and mortality in epidemiological studies [2]. Flavonoids, secondary metabolites in plants, are suggested to be among the bioactive compounds in fruit and vegetables that contribute to the cardiovascular benefits [3]. Flavonols, particularly quercetin and kaempferol, are among the most widely distributed flavonoids in foods [4,5]. Quercetin and kaempferol have been shown to have antioxidant and anti-inflammatory effects in in vitro studies [6] and cardioprotective and antihypertensive benefits in animal studies [7,8]. Flavonols in plants, however, are synthesized linked to sugar conjugates, and thus have different bioavailability than the free forms [9]. Little is known about the potential cardiovascular effects in humans when foods rich in flavonols are consumed.

For flavonols to exert bioactivity in humans, ingested flavonols need to be bioavailable and reach body tissues. Bioavailability from a nutritional perspective is defined as the extent of digestion, absorption, metabolism, and excretion of a compound after the ingestion of food [10]. Establishing the bioavailability of bioactive compounds is an essential step in determining the potential mechanisms of action of flavonols [10]. Human studies evaluating the bioavailability of quercetin and kaempferol suggest that some conjugated forms in plants have higher bioavailability than the free forms [11,12,13]. In the human body, flavonols are rapidly metabolized which has limited the detection of the free forms in blood and urine and the evaluation of bioactivity of flavonol-rich foods [14]. However, recent advancements in mass spectrometry enable the detection of low-abundance metabolites [15]. The bioactivity of circulating metabolites in humans is not well understood. A better understanding of the bioavailability of flavonol conjugates from different food sources is needed to inform clinical studies. This article aims to review the current literature on the bioavailability of quercetin and kaempferol from food sources and to evaluate the potential cardiovascular effects in humans.

## 2. Food Sources and Dietary Intakes

Quercetin and kaempferol are widely distributed in fruit and vegetables [16]. Table 1 shows the average amounts in select major food sources. High concentrations of quercetin are found in a few foods such as onion, asparagus, and berries, and small quantities are found in many different fruit and vegetables. The richest plant sources of kaempferol (mg/100 g fresh weight) are green leafy vegetables, including spinach and kale, and herbs such as dill, chives, and tarragon. The leaves of wild leeks or ramps (100g fresh weight) were reported to contain 50.2 and 32.5 mg of quercetin and kaempferol, respectively [17].

The estimated dietary intakes of flavonols vary across populations. Differences are related, in part, to variations between databases. Currently, there is not a complete standard database on flavonoid content in foods. Dietary recommendations of flavonol intake for individuals have not been established [18,19]. Despite being widely available in foods, flavonol intake in the US has been reported to range between 9.0–36.2 mg/day [20] which is lower than the 51 and 52 mg/day intake reported in the United Kingdom and European countries respectively [21,22]. In the US, the foods that contributed most to flavonol intake are tea, onion, apple, and red wine [23,24]. Average intakes of individual quercetin and kaempferol among US adults are 3.5 and 5.4 mg/day, respectively [23]. 

## 3. Chemical Structure

**Figure 1:**
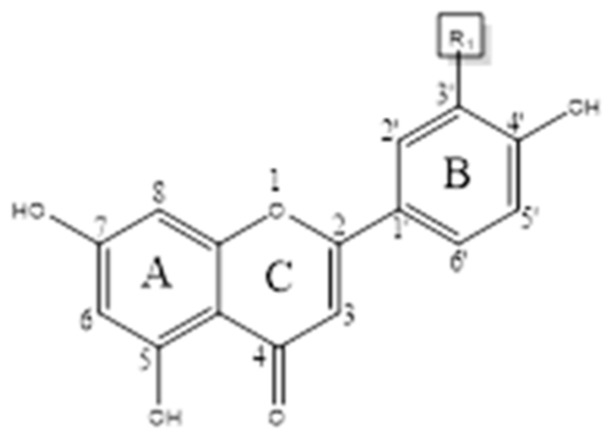
General structure of quercetin and kaempferol. Two phenyl rings (A and C) and a heterocyclic ring B. If R_1_ = OH, Quercetin; if R_1_ = H, Kaempferol.

Flavonoids consist of two phenyl rings (ring A and B) connected to a heterocyclic ring (ring C) [25]. Quercetin and kaempferol share the same 3-hydroxy flavone backbone but differ by the presence of an additional hydroxyl group at the R1 position on quercetin [26] (Figure 1). The number of hydroxyl groups influences the chemical reactivity [26] of the compounds. Thus, kaempferol is more chemically stable and less reactive than quercetin as it has one less hydroxyl group. Flavonols in their free forms, aglycones, have lipophilic (fat-soluble) properties. However, most flavonols are synthesized in plants are attached to a sugar moiety, the glycoside form, which are lipophobic (water-soluble) [27]. The hydroxyl functional groups on all three rings are potential sites for linkage to sugar moieties (i.e., O-glycosides) [25]. The sugar moieties most commonly attached to flavonols are monosaccharides glucose, rhamnose, galactose, arabinose, and xylose [28] and the disaccharide rutinoside which is composed of glucose and rhamnose connected by a β-glycosidic bond [27]. 

Table 2 shows the major quercetin and kaempferol glycosides reported in plant sources. Dietary quercetin is present mainly as O-glycosidic forms including quercetin-3-O-rutinoside (rutin), quercetin-3-O-glucoside (isoquercetin), and quercetin-3,4′-O-diglucoside [29]. The specific quercetin moieties in apples, a main source of intake in the US, are mainly -rutinoside, -galactoside, -rhamnoside, and -glucoside [30]. The position of conjugate attachment may block the active -OH sites that contribute to the potency or bioactivity of the compounds [25]. Also, variations of sugar moieties synthesized in plants result in different rates of digestion, absorption, and metabolism [31]. Thus, high amounts of quercetin and kaempferol in foods does not always lead to increased bioactivity.

## 4. Bioavailability 

**Figure 2:**
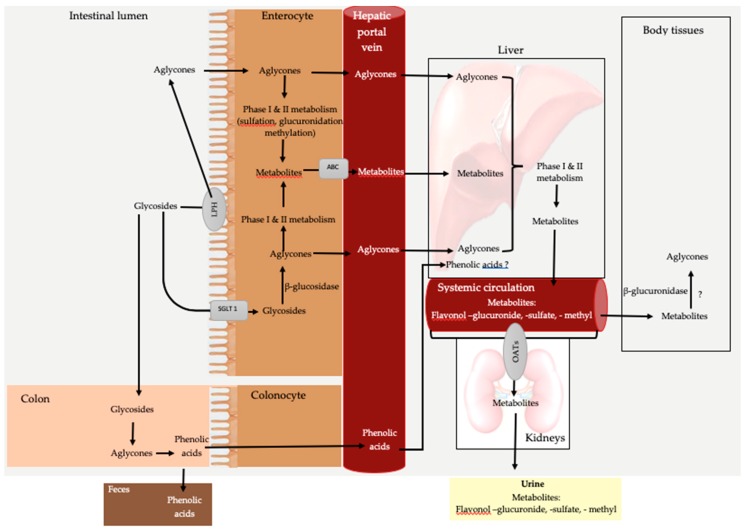
General overview of dietary flavonol bioavailability. Abbreviations: LPH, lactase-phlorizin hydrolase enzyme; SGLT 1, sodium-dependent glucose transporter; ? = mechanism is not well understood.

Figure 2 illustrates the bioavailability of dietary flavonols after the ingestion of aglycone and glycoside forms. One of the important factors of bioavailability is the fat solubility of the ingested flavonols [10]. When flavonol-rich foods are ingested, the aglycones and glycoside forms undergo different routes of digestion and absorption [40]. Lipophilic aglycons passively diffuse unmetabolized from the intestinal lumen into the enterocytes where they are either directly absorbed into the hepatic portal vein or metabolized before absorption [40,41]. Metabolism of the aglycones in the enterocytes involves phase I (oxidation and O-demethylation) and phase II metabolism (sulfation, glucuronidation, and methylation) to produce metabolites which are absorbed via ATP-binding cassette (ABC) transporters into the hepatic portal vein [41,42]. 

Lipophobic glycosides, however, must be hydrolyzed to the aglycone form in the intestinal lumen or enterocyte before they can be absorbed into the blood [43]. On the intestinal brush border, lactase-phlorizin hydrolase enzyme (LPH) hydrolyzes glycosides to aglycones [44] which are passively absorbed into enterocytes. Alternatively, the glycosides can be transported by sodium-dependent glucose transporter (SGLT 1) into the enterocyte where they are hydrolyzed by cytosolic β-glucosidase [43,45]. The resulting aglycones then either passively diffuse into the hepatic portal vein or undergo phase I and II metabolism to produce metabolites which are absorbed via ABC transporters [41,42] into the hepatic portal vein. The absorbed aglycones bound to serum albumin and the metabolites are transported to the liver [46]. In the liver, the remaining aglycones undergo phase I and II metabolism resulting in methyl, sulfur, and glucuronide metabolites which are transported along with intestinal metabolites into the systemic circulation for distribution to body tissues [47,48]. Flavonol metabolism in body tissues is not well understood. An in vitro study suggested that β-glucuronidase enzyme found in body tissues hydrolyzes the conjugated metabolites producing aglycones [49]. Flavonol metabolites are excreted by urinary and biliary elimination. Flavonols are transported from blood circulation to the kidney via organic-anion-transporting polypeptides (OATs) [50,51]. The OATs transporters are specific for the transport of metabolites synthesized endogenously in the liver (sulfate, glucuronide, and methyl metabolites) [51]. Metabolites in bile are either eliminated in feces or recycled back to the small intestine [52]. Some ingested glycosides are poorly absorbed in the small intestine and reach the colon where the colonic microbiome metabolism occurs [48]. The major metabolites produced are 3,4-dihydroxyphenylacetic acid, 3,4-dihydroxybenzoic acid, and 3-hydroxyphenylacetic acid [53]. These metabolites are either excreted in feces or absorbed into blood circulation [20,54]. The fate and bioactivity of phenolic acid metabolites are not well understood.

### 4.1. Quercetin Bioavailability 

#### 4.1.1. Digestion and Absorption

Table 3 shows quercetin absorption reported from different food sources. deVaries et al. [55] compared the absorption of quercetin from onions and tea. Healthy participants followed a standard low-quercetin diet (vegetables and fruit <15 mg quercetin/kg and beverages <4 mg/L) during the study period. They consumed 1600 mL/day of black tea (49 mg quercetin glycosides) and 129 g/day of fried onions (13 mg quercetin glycosides) each for three days separated by a four-day washout period. The washout period was sufficient to decrease urinary quercetin levels to baseline. Urinary excretion was higher after the intake of fried onions than black tea (1.1% versus 0.5%) suggesting that the form of quercetin from onions is better absorbed than the form in tea. The deVaries group [56] then compared the quercetin absorption from six 125 mL glasses of red wine (14.2 mg quercetin), 50 g of fried yellow onions (15.9 mg quercetin), and three 125 mL cups of black tea (13.7 mg quercetin). Each was consumed daily for four days separated by 3-day washout periods. The washout period was sufficient to decrease plasma levels to baseline. Plasma concentration of quercetin was higher after onion intake than red wine or tea, and 24-h urinary excretion was highest after onion intake followed by red wine and tea. Although tea and red wine are rich sources of quercetin, the form of sugar moieties had a significant effect on absorption. Notably, the only difference between some glucosides in onions and red wine is the position of the glucose attachment (Table 2). 

Olthof et al. [57] tested whether the position of glucose moiety affected absorption. Participants consumed capsules containing 151 mg quercetin-3-glucoside and 154 mg quercetin-4′-glucoside. The difference in 24-h urinary excretion was 3.0% and 2.6% (*p* > 0.05), respectively, with no difference in plasma peak concentrations between treatments. Thus, the glucose attachment on positions 3′ and 4′ had no impact on the rate of absorption. This can be explained by the suggested mechanism of transport in the small intestine. Quercetin glucoside is transported by the sodium-dependent glucose transporter (SGLT 1) into enterocytes independent of its attachment to quercetin [41]. 

Hollman et al. [58] compared absorption from fried onions (225 µmol quercetin), applesauce with peels (325 µmol), and a quercetin-3-O-β-rutinoside or rutin (331 µmol) capsule in nine healthy participants. The excretion of free quercetin in the urine was 1.39% for onions, 0.44% for applesauce and 0.35% for rutin. Plasma peak levels were reached at 0.7 h after eating onions, 2.5 h after applesauce, and 9 h after rutin. Absorption was highest with onions compared to applesauce and rutin supplements. Although quercetin content in apples is concentrated in the peels, the high amounts of insoluble fiber in apples’ skin may interfere with intestinal absorption [62,63]. Quercetin rutinoside, the major glycoside in tea and apples, is a disaccharide connected by a β-glycosidic bond. Humans lack the enzyme needed to hydrolyze this bond. Consequently, microorganisms in the colon mediate hydrolysis of the rutinoside resulting in minimal intestinal absorption and production of phenolic acid metabolites in the colon.

Hollman et al. [11] compared the absorption of quercetin from onions, rutin, and aglycones with the exclusion of microbiome metabolism to quantify quercetin absorption from the small intestines only. To eliminate colonic absorption, the study was conducted on healthy ileostomy participants (*n* = 9) who consumed 89 mg of quercetin from fried yellow onions, 220 mg β-rutinoside in capsules and 112 mg aglycone from quercetin dehydrate capsules for four days. Urine and ileostomy effluent were used to calculate percent absorption. The average absorption was 52% for fried onions, 24% for pure aglycone, and 17% for rutinoside. This suggested that quercetin glucoside from onions has the highest extent of intestinal absorption relative to quercetin rutinoside and aglycone. This can be explained by the different routes of absorption across the intestinal wall. Quercetin glucosides are absorbed by SGLT 1 which is an active transporter that requires energy for its action and thus has a higher absorption rate than the passive transport of the aglycones across the intestinal wall.

Lipid solubility is a major determinant factor in absorption. The hydrolysis of a sugar moiety before absorption into the bloodstream increases lipid solubility of the ingested quercetin. Therefore, it is important to examine the role of dietary fat on the rate and extent of absorption. Guo et al. [60] conducted a crossover study in which participants consumed 1095 mg quercetin aglycone supplement in muffins that were fat-free (<0.5 g fat), low-fat (4.0g) or high-fat (15.4 g). Maximum concentration of total plasma quercetin increased by 12% after the low-fat trial and increased by 45% after high-fat trial (*p* < 0.05). Enhanced quercetin absorption after the high-fat trial can be explained by increased incorporation into the micelle, soluble fat droplet, indicating that the co-ingestion of quercetin with dietary fat increases absorption from the small intestine. 

#### 4.1.2. Metabolism and Excretion

After absorption, quercetin is transported to the liver where it undergoes phase I and II metabolism producing metabolites which circulate in the blood for distribution to body tissues [46]. To understand quercetin bioavailability, it is essential to identify the major metabolites in the blood and urine. Table 4 lists quercetin metabolites detected in the blood and urine after the ingestion of flavonol-rich food. Mullen et al. [64] analyzed and quantified major quercetin metabolites in plasma and urine after ingestion of onions. Healthy participants followed a low-quercetin diet for two days and fasted overnight before the consumption of 270 g fried onions. Venous blood samples were collected before onion intake and 0.5, 1, 2, 3, 6, and 24 h post-ingestion. The three major plasma metabolites were quercetin-3-sulfate, -3′-sulfate, and -3-glucuronide. The main 24-h urinary metabolites were quercetin -diglucuronide, -3′-glucuronide, isorhamnetin-glucuronide, –glucuronide sulfate, and -methyl quercetin diglucuronide. In total, 23 metabolites were identified with five being quantified in plasma and 12 in the urine.

Quercetin metabolites appeared in plasma after 30 min of ingestion, but a significant amount was excreted over a 24-h period. This indicates rapid clearance and a short half-life of quercetin in the blood. To understand the accumulation of quercetin in plasma after multiple administrations of quercetin-rich foods, Moon et al. [65] determined quercetin conjugate accumulation in human plasma after the periodic ingestion of onions. Participants (*n* = 7) consumed 93.6 mg quercetin/day from onion slices over three meals for one week. Glucuronide and sulfate metabolites in fasting plasma increased from 0.04 μM to 0.63 μM (*p* < 0.05). This was the first human study to report that short-term ingestion of quercetin glucosides in onions elevates plasma metabolites and accumulates even after fasting. The highest concentration of quercetin metabolites was detected after the ingestion of onions. Major plasma metabolites are quercetin-3′-sulfate and -3-glucuronide with maximum levels reached after 0.8 and 0.6 h, respectively. In addition, major urine metabolites are quercetin -diglucuronide, -3′-glucuronide, isorhamnetin-3-glucuronide, and -glucuronide sulfate, and these reached maximum levels after 4 h [64]. This indicates that the kidney plays a role in quercetin metabolism. Kidney metabolism includes the addition of glucuronide and sulfate conjugates on different sites on quercetin structure. Notably, small amounts of glucoside metabolites were detected in urine but not in the blood. 

In summary, quercetin glucosides from onions appear to have the highest rate of absorption compared to the glycosides from apples, red wine, and tea or aglycones. In addition, dietary fat has been shown to enhance quercetin aglycone absorption from the small intestine. The major plasma metabolites are quercetin-3′-sulfate and -3-glucuronide with maximum levels reached after 0.8 and 0.6 h, respectively. Major urine metabolites are quercetin -diglucuronide, -3′-glucuronide, isorhamnetin-3-glucuronide, and -glucuronide sulfate reached maximum levels after 4 h. Quercetin in plasma was only detected as metabolites, and thus further studies are needed to investigate the bioactivity of quercetin metabolites.

### 4.2. Kaempferol Bioavailability

#### 4.2.1. Digestion and Absorption

Studies evaluating the bioavailability of food-derived kaempferol conjugates are limited. In a crossover study, De Varies et al. [55] examined the digestion and absorption of kaempferol from black tea in participants (*n* = 15) who consumed 27 mg of kaempferol from black tea for three days. Urinary excretion of kaempferol was 2.5% of the amount ingested suggesting that kaempferol absorption was higher than quercetin (0.5% urinary excretion). This indicated that although quercetin content is higher than kaempferol in black tea, the type of glycoside in tea had higher bioavailability.

The digestion and absorption of kaempferol were assessed after the intake of 12.5 mg kaempferol from broccoli for 12 days. The rate of kaempferol urinary excretion was 0.9% [66]. In rats, it was suggested that kaempferol could be converted to quercetin by phase I oxidation enzymes [67]; however, no quercetin was detected after the ingestion of broccoli indicating that quercetin cannot be endogenously synthesized from dietary kaempferol in humans. This was confirmed in another study which examined the rate of digestion and absorption of kaempferol after intake of 9 mg kaempferol from cooked endive [13]. The 24-h urinary excretion of kaempferol was 1.9% and the plasma peak concentration reached 0.1 µM after 5.8 h. No quercetin was detected in plasma or urine. Endive contains the glucuronide form which is one of the metabolites released after phase I and II metabolism. This metabolite can be absorbed by the ABCs transporters and thus has higher absorption; but, the rate and efficiency of this transport mechanism are not well defined. 

#### 4.2.2. Metabolism and Excretion

Generally, flavonols are extensively metabolized in the liver and circulate in the blood as sulfate, methyl, and glucuronide conjugates [64]. Identifying the major metabolites in blood and urine after the ingestion of kaempferol-rich foods is needed to better understand the metabolites’ potential biological activity. Only one human study identified kaempferol metabolites after the ingestion of kaempferol-rich food. DuPont et al. [13] assessed kaempferol metabolites after the ingestion of 150 g of cooked endive (9 mg kaempferol). The major metabolite identified in plasma and urine was kaempferol-3-glucuronide. In addition, kaempferol mono- and di-sulfates were detected in urine. Although studies on quercetin metabolism did not detect aglycone due to rapid metabolism, in this study, free kaempferol was detected in plasma and urine (40% and 16% of total kaempferol, respectively). This result can be explained by the activity of the β-glucuronidase enzyme which hydrolyzes glucuronide metabolites in body tissues [68]. It was suggested that the activity of this enzyme is higher for kaempferol-3-glucuronide compared to quercetin glucuronide which may explain the absence of quercetin aglycone in plasma and urine [68]. The concentration of free kaempferol was lower in urine than blood suggesting that some of the aglycones were metabolized in the kidney before excretion. 

In summary, an evaluation of the literature on kaempferol digestion and absorption indicated that kaempferol rutinoside and glucoside in tea have the highest absorption, followed by glucuronide and glucoside in endive and sophoroside in broccoli. Kaempferol metabolites were identified in the plasma as aglycone and glucuronide and as sulfate in the urine; however, before conducting intervention studies on the health benefits of kaempferol, further human studies are needed to assess the absorption of kaempferol from foods. 

## 5. Potential Bioactivity in Humans 

### 5.1. Quercetin Bioactivity

Clinical trials on the bioactivity of quercetin-rich foods or diets on blood pressure and cardiovascular risk are limited (Table 5). Conquer et al. [71] reported on the first clinical trial to study the effect of quercetin (1 g quercetin aglycone with 200 mg rutin in a capsule versus a placebo) on plasma quercetin concentrations and CV risk factors. Quercetin treatment significantly increased the plasma concentration from 0.1 µmol/L to 1.5 µmol/L after 28 days, but no significant changes in CV or thrombogenic risk factors (i.e., platelet aggregation, platelet thromboxane production, blood pressure or resting heart rate) between groups were detected. Participants were healthy, normotensive individuals which may explain the lack of treatment effect. Edwards et al. [72] found that 730 mg quercetin aglycone was effective in reducing blood pressure in patients diagnosed with stage 1 hypertension, but not in pre-hypertensive individuals. Another study found that a lower dose of 150 mg quercetin aglycone was effective in reducing blood pressure in patients with hypertension after 42 days [73]. Participants were instructed to continue antihypertensive medications (*n* = 15). No significant changes were reported in oxidative stress markers or inflammatory markers including tumor necrosis factor-alpha (TNF-α) and CRP. Conversely, intake of 500 mg of quercetin significantly decreased inflammatory markers, TNF-α and interleukin (IL-6), in women with type 2 diabetes, a risk factor for CVD [74]. 

Quercetin supplements supply the aglycone form which is not the most bioavailable. Based on the bioavailability studies reviewed, quercetin glucoside was the most bioavailable form. It is plausible that it may be effective at lower doses than the aglycone form. Brüll et al. [75] conducted a double-blind placebo-controlled crossover trial to investigate the effects of onion extract on blood pressure in adults with pre- and stage 1 hypertension who were in proinflammatory state (hs-CRP ≥2 mg/L). Participants taking antihypertensive medications (*n* = 12) continued medications. Quercetin capsules of 132 mg onion skin extract (162 mg quercetin) and a placebo were administered daily for six weeks separated by a six weeks washout period. In the whole group, quercetin did not significantly decrease 24-h ambulatory blood pressure parameters. However, in the subgroup with stage 1 hypertension, quercetin significantly decreased 24-h systolic blood pressure by 3.6 mmHg, day-time systolic blood pressure by 4.6 mmHg, and night-time systolic blood pressure by 6.6 mmHg. Fasting serum intercellular adhesion molecule decreased by 8.2 ng/mL, but no significant difference was detected in other indicators of vascular damage and inflammation.

The significant reduction in blood pressure among the stage 1 hypertensive subgroup agreed with previous studies which indicated a threshold for quercetin effectiveness. Also, a meta-analysis review evaluated the effectiveness of quercetin supplement in lowering blood pressure in 7 trials. Results showed that a dosage of ≥500 mg quercetin aglycone supplement significantly reduced systolic and diastolic blood pressure by 4.45 mmHg (*p* < 0.007) and −2.98 mmHg (*p* < 0.001), respectively [76]. The evaluation of the impact of quercetin on oxidative stress and vascular function markers suggested that quercetin effects on blood pressure may be independent of endothelial function and angiotensin converting enzyme (ACE) mechanism which agreed with Larson et al. [77] findings that reported 1095 mg of quercetin supplements decreased blood pressure independently to ACE and other vascular damage markers. 

Information contained in Table 4 indicated that quercetin supplements as low as 150 mg of aglycone were effective in lowering blood pressure in individuals with stage 1 hypertension independent of ACE activity, oxidative stress, and vascular damage markers. A dose of 500 mg was effective in lowering inflammatory markers, TNF-α and IL-6. Further studies are needed to investigate the bioactivity of quercetin metabolites after the ingestion of glucosides from quercetin-rich foods on inflammatory markers in patients with elevated markers and at high risk of CVD. 

### 5.2. Kaempferol Bioactivity

The potential cardioprotective effects of kaempferol in in vitro and animal studies have been attributed to its anti-inflammatory activities [6,79,80]. Epidemiological studies have investigated associations between intakes of dietary flavonoids, specifically kaempferol, and cardiovascular health (Table 6), but clinical trials on the cardioprotective benefits of kaempferol are limited. The bioactivity in humans depends on the type of ingested conjugates and their bioavailability. There is a general lack of data on dietary kaempferol bioavailability and absorption in humans. No clinical trials were identified on the cardiovascular bioactivity in humans. 

The first study to evaluate the correlation between the intake of flavonoid-rich foods and mortality from coronary heart disease (CHD) was a longitudinal study in a prospective cohort of 805 men [81]. Participants’ average flavonoid intake was 25.9 mg/day. The consumption of flavonoids rich food was inversely associated with mortality from CHD (95% CI; 0.20–0.88, *p* = 0.015). This agrees with the findings of a meta-analysis review of the association between flavonol intake and risk of CHD mortality [82]. The review concluded that a 20% reduction in CHD mortality rate was observed among individuals in the highest tertile of flavonol intake. The mean daily flavonol intake ranged between 2 and >34 mg, mainly from tea, onions, apples, and broccoli. However, the association of individual flavonols was not analyzed.

An assessment of the association between the intake of individual flavonols and myocardial infarction (MI) and fatal CHD in the Nurses’ Health study indicated that kaempferol intake, mainly from broccoli and tea, was inversely associated with CHD with a relative risk of 0.66 (95% CI: 0.48–0.93, *p* = 0.04), but no significant association was observed for MI [83]. However, two studies reported a significant negative association between acute and fatal MI with higher flavonol and kaempferol intake [84,85]. To assess whether kaempferol is associated with reduced inflammation in humans, Bobe et al. [86] investigated the association between kaempferol and IL-6 levels, an inflammatory marker, in participants with elevated inflammation. Results showed that kaempferol was significantly associated with lower IL-6 level among participants with the higher dietary intake (>21.4 mg/day).

The reviewed studies indicate that a daily intake of kaempferol ≥1.5 mg/day was associated with lower CHD mortality and MI incidence. This potential cardioprotective benefit is inconclusive due to several limitations. The intake of kaempferol was mainly from vegetables, fruit, and tea which may contain other bioactive compounds that contribute to cardioprotective effects. The estimated intake of kaempferol is inaccurate due to the absence of a complete database and variations of kaempferol content in foods between studies. Intervention studies on the effects of kaempferol derived from plant sources are still needed to confirm its cardioprotective benefits in humans.

## 6. Safety 

### 6.1. Quercetin Safety 

Clinical studies were reviewed to evaluate the potential adverse effects of quercetin in Table 7. Oral quercetin was mainly administered as a purified aglycone supplement in human studies. The supplemented dosage ranged between 150–5000 mg/day for a maximum duration of 12 weeks. Quercetin metabolism mainly occurs in the liver, and metabolites are excreted by the kidneys. Only two human studies were identified that assessed quercetin safety on liver or kidney biomarkers. Egert et al. [73] examined the safety of 150 mg/day quercetin aglycone intake for six weeks in overweight and obese participants at a high risk of CVD. Liver and kidney biomarkers measured were alanine transaminase, aspartate transaminase, g-glutamyl-transpeptidase, alkaline phosphatase, cholesteryl esterase, and creatinine. Additionally, hematology (i.e., leucocyte, erythrocyte, platelet count, and hemoglobin concentration) and electrolytes were measured. No significant change in liver, kidney, hematology, or electrolytes biomarkers was detected at the end of the treatment period indicating a daily dose of 150 mg was safe. In a phase I dose-escalating study evaluating the safety of quercetin in patients with untreated chronic hepatitis C, doses administered ranged 250–5000 mg/day (*n* = 2–3 per dose group) for 28 days. Results showed that all participants tolerated quercetin without changes in liver enzymes (i.e., alanine and aspartate transaminases), and blood count, complete metabolic, and cholesterol panels remained unchanged. A few patients experienced mild gastrointestinal discomfort, but the actual number and doses were not reported. However, the safety results from the study are inconclusive because of the sample size was very small in each dose group, and the participants had liver disease which may have altered quercetin metabolism. Although few clinical studies investigated the effectiveness of quercetin supplements report on safety measures. It was found that amounts as high as 5000 mg/day supplemented for 4 weeks did not cause adverse events. In 2010, quercetin supplements were added to the Food and Drug Administration’s Generally Recognized as Safe (GRAS) list for use as a supplemental ingredient added in foods and beverages up to 500 mg per serving [88].

### 6.2. Kaempferol Safety

No human trials were identified that reported the potential toxicity or adverse events of oral kaempferol intake. Although in vitro studies reported kaempferol antioxidative effects, high levels of kaempferol supplement may cause self-oxidation (pro-oxidation) [92,93]. However, animal studies found that after oral intake, no pro-oxidation effect was observed [94]. A few in vitro studies found that kaempferol decreases iron absorption and cellular uptake of folic acid due to its high reactivity with these nutrients [95,96]. However, average kaempferol amount reported in US diet was 5.4 mg/day and consumption of kaempferol-rich foods providing 8.04 mg/day was associated with beneficial effects with no reported adverse events [23,86].

## 7. Conclusions

Based on the current evidence, the most bioavailable form of quercetin is the glucoside conjugate which is mainly found in onions. A few human studies investigated kaempferol bioavailability and reported that kaempferol glucoside and rutinoside in tea were the most bioavailable forms. Once absorbed, quercetin and kaempferol are rapidly metabolized in the liver to form glucuronide, methyl, and sulfate metabolites which can be detected in the blood and urine. Therefore, the bioactivity and metabolism of quercetin and kaempferol metabolites in body tissues need to be investigated to better understand the mechanism of action on cardiovascular health. The optimal effective dose of quercetin reported to have beneficial effect of lowering blood pressure and inflammation is 500 mg of aglycone which was found to be a safe dose. Little is known about kaempferol potential cardioprotective benefits. Studies are needed to evaluate the potential cardiovascular benefits of plants rich in quercetin and kaempferol glycoside conjugates.

## Figures and Tables

**Table 1:** Select Plant Sources of Quercetin and Kaempferol.

Source	Quercetin	Kaempferol
Food	mg/100 g fresh weight	
Apples	4.01	0.14
Asparagus	14.0	1.40
Broccoli	13.7	7.20
Chili pepper	32.6	-
Chinese cabbage	-	22.5
Kale	22.6	47.0
Leeks	0.9	2.67
Lettuce	14.7	0.84
Onions	45.0	4.50
Spinach	27.2	55.0
Chives	10.4	12.5
Dill	79.0	40.0
Fennel leaves	46.8	6.50
Oregano	42.0	-
Blueberry	14.6	3.17
Cherry	17.4	5.14
Cranberry	25.0	0.21
Wild leeks (whole) [17]	8.36	5.31
Beverage	mg/100 ml	
Black tea	2.50	1.70
Red wine	3.16	0.25

Sources: Phenol-Explorer and USDA (United States Department of Agriculture) Database for the Flavonoid Content of Selected Foods.

**Table 2:** Major quercetin and kaempferol glycosides in select plant sources.

Food Source	Sugar Moieties	Ref.
Quercetin		
Red wine	-3-glucoside, –rutinoside	[32]
Onions	-4′-glucoside, -3,4′-diglucoside	[33]
Tea	-3-rutinoside	[34]
Apple	-rutinoside, -galactoside, -rhamnoside, –glucoside	[30]
Wild leek (ramps)	-sophoroside glucuronide, -hexoside glucuronide, -sophoroside	[17]
Kaempferol		
Wild garlic and leeks	-glucopyranoside, -neohesperidose	[35,36,37,38]
Black tea	-rutinoside, glucoside	[27]
Broccoli	-sophoroside (β-1,2-glucose)	[39]
Endive	-3- glucuronide, 3-glucoside	[13]
Wild leek (ramps)	-sophoroside glucuronide, -rutinoside glucuronide, -sophoroside	[17]

**Table 3:** Extent and rate of digestion and absorption different of quercetin forms from dietary sources.

Food Source (amount, g or ml)	Quercetin Dosage	*n*	Urinary Excretion or Concentration (%)	Maximal Plasma Concentration (µmol/L)	Time to Reach Maximal Concentration (hours)	Ref.
Black tea (1600)	49 mg	15	0.5			[55]
Onions (129)	13 mg		1.1			
Onions (NR)	225 µmol	9	1.39	0.74	0.70	[58]
Applesauce + peel (NR)	325 µmol		0.44	0.30	2.5	
Rutin	331 µmol		0.35	0.30	9	
Onions (333)	89 mg	9	52			[11]
Rutin	220 mg		17			
Dehydrate	112		24			
Red wine (750)	14.2	12	0.371 μmol/L	0.026		[56]
Onions (50)	15.9		0.509 μmol/L	0.053		
Black tea (375)	13.7		0.252 μmol/L	0.026		
Quercetin-3-glucoside capsule	151	9	3.0	5.0	0.62	[57]
Quercetin-4′-glucoside capsule	154		2.6	4.5	0.45	
Dehydrate	544	6	1.69 μmol/L			[59]
Onion soup (100)	47		1.17 μmol/L			
Dehydrate with fat-free (<0.5)	1095	9	-	1.1	5.7	[60]
Dehydrate low-fat (4.0)			-	1.24	5.4	
Dehydrate high-fat (15.4)			-	1.6	6.7	
Onions (160)	100	12	6.4	2.31	0.68	[61]
Dehydrate	100		4.5	2.12	0.70	
Buckwheat tea (NR)	200		1.0	0.64	4.32	
Dehydrate	200		0.90	0.32	6.98	

Abbreviations: NR, not reported; min, minutes; *n*, number of participants.

**Table 4:** Major quercetin metabolites in blood and urine after the ingestion of quercetin-rich foods.

Food Source (g or ml)	Flavonol Glycoside (mg)	Dosage (mg)	Metabolites Detected	Concentration (µmol/L)	Max Time (hours)	Half-life (hours)	Ref.
Blood							
Fried onions (200)	Quercetin-3,4′-O-diglucoside	37.1	Isorhamnetine	0.11	1.5	-	[69]
	Quercetin-3-O-glucoside	0.7	-glucuronide	0.26	1.5	-	
	Quercetin-4′-O-glucoside	39.5	-Sulfate	0.16	1.5	-	
	Isorhamnetin-4′-O-glucoside	1.8					
	Quercetin	0.1					
Fried onions (270)	Quercetin-3,4′-O-diglucoside	67	-3′-sulfate	0.67	0.75	1.71	[64]
	Quercetin-4′-O-glucoside	66	-3-glucoronide	0.35	0.60	2.33	
	Isprhamnetin-4′-O-glucoside ^*^	5.3	Isorhamnetin-3-glucuronide^*^	0.11	0.60	5.34	
			-glucuronide sulfate	0.12	2.5	4.54	
			-diglucuronide	0.062	0.80	1.76	
Tomato juice (300)	Quercetin-3-O-rutinoside	7.3	-3-glucuronide	0.0038	5	5.7	[70]
Urine							
Fried onions (270)	Quercetin-3,4′-O-diglucoside	67	-diglucuronide	2.22	4-8	-	[64]
			quercetin-3′-glucuronide	1.85	0-4	-	
	Quercetin-4′-O-glucoside	66	isorhamnetin-3-glucuronide^*^	1.79	4-8	-	
			-glucuronide sulfate	1.38	0-4	-	
			Methylquercetin diglucuronide	1.00	4-8	-	
			-3-glucuronide	0.912	0-4	-	
	Isorhamnetin-4′-O-glucoside ^*^	5.3	-glucoside sulfate	0.82	0-4	-	
			Isorhamnetin-4′-glucuronide^*^	0.70	0-4	-	
			-glucoronide glucoside	0.16	0-4	-	
			-4′-O-glucuronide	0.24	24	-	
Tomato juice (300)	Quercetin-3-O-rutinoside	7.3	-3-glucuronide	0.18	24	-	[70]

* Isorhamnetin indicates quercetin is connected to methyl.

**Table 5:** Studies on the effectiveness of quercetin on hypertension, inflammation, and cardiovascular risk.

*n*	Health Condition	Age (years)	BMI (kg/m^2^)	Quercetin (mg)	Duration (days)	Blood Pressure Results	Other Results	Ref.
27	Healthy	42.0 ± 2.6	26.0 ± 1.3	1000 aglycone + 200 rutin	28	No effect	No effect other CVD factors	[71]
41	Pre-HTN	47.8 ± 3.5	29.7 ± 1.3	730 aglycone	28	No effect	nor oxidative stress	[72]
	Stage 1-HTN	49.2 ± 2.9	29.7 ± 1.3			SBP (−7 ± 2mmHg), DBP(−5 ± 2mmHg) mean arterial pressures (−5 ± 2 mmHg)	no effect on oxidative stress	
93	Baseline mean BP (130±16.4/ 81.6±9.3mmHg)	25–65	25–35	150 aglycone	42	SBP: entire group (−2.6 mmHg), subgroup with HTN (−2.9 mmHg) Subgroup aged 25-50 (−3.7 mmHg)	Decreased oxidized LDL. No effect on TNF-α and CRP	[73]
62	Type 2 diabetes	35-55	NR	500 aglycone	70	SBP (−8.8 ± 9.3 mmHg), DBP (no effect)	Decreased TNF-α and IL-6 relative to baseline, but not different than placebo	[74]
68	Pre-HTN (≥120–139 mmHg and/or ≥80–89 mmHg)	25–65	25–35	396 onion powder (162 quercetin glucoside)	42	ABP (no effect)	sICAM-1 (−8.2 ng/mL)	[75]
	Stage I HTN (≥140–159 mmHg and/or ≥90–99)	25–65	25–35			Systolic ABP (−3.6 mmHg),	sICAM-1 (−8.2 ng/mL) No effect on NO, ACE, sVCAM	
5	Normotensive	24 ± 3	24 ± 4	1095 aglycone	1	No effect	No effect on ACE, ET-1, NO, and brachial artery flow mediated dilation	[78]
12	Stage 1 HTN	41 ± 12	29 ± 5			SBP (−5 mmHg)		

Abbreviations: NR, not reported; HTN; hypertension; BMI, body mass index; CVD, cardiovascular disease; BP, blood pressure; SBP, systolic blood pressure; DBP, diastolic blood pressure; ABP, ambulatory blood pressure; LDL, low-density lipoprotein; TNF-α, tumor necrosis factor-alpha; CRP, c-reactive protein; IL-6, Interleukin 6; sICAM-1, soluble intercellular adhesion molecule-1; sVCAM, circulating vascular cell adhesion molecule-1; ACE, angiotensin-converting enzyme; NO, nitric oxide; ET-1, Endothelin-.

**Table 6:** Epidemiological studies on the association between flavonoids intake and cardiovascular risk.

*n*	Age (years)	Health Status	Dietary Intake (mg/day)	Results	Ref.
805	65–84	Healthy	Flavonoids (12.0–41.6)	(-): CHD mortality (0.42, 95% CI 0.20–0.88, p = 0.015)	[81]
4807	64–69	Healthy	Flavonols (14.8–38.5)	(-): fatal MI (0.57, 95% CI 0.33–0.98)	[84]
66,360	30–55	29.8% HTN	flavonols+flavone (21.2) Kaempferol (4.7)	(-): CHD mortality (0.66, 95% CI 0.48–0.93; p = 0.04) No association with MI	[83]
10,054	Mean 39.3	9.6% HTN	flavonoids (24.2), kaempferol (0.1–0.9)	(-): cerebrovascular disease (0.70, 95% CI 0.56–0.86, p = 0.003), thrombosis (0.63, 95% CI 0.47–0.85, p = 0.004)	[87]
872	52–67	Colorectal adenoma	Flavonols (9.4–20.8), kaempferol (2.54–8.04)	(-): IL-6 levels (95% CI: 0.24–0.93; p = 0.03)	[86]
744	65–99	Healthy	Kaempferol (1.0–1.5)	(-): acute MI (0.48, 95% CI; 0.30–0.77, p = 0.002).	[85]

Abbreviations: BMI; body mass index, (-); inverse association with flavonol or kaempferol intake, CHD; coronary heart disease, MI; myocardial infarction, USA; united states of America, HTN; hypertension, DM; diabetes mellitus, IHD; ischemic heart disease, NR; not reported.

**Table 7:** Summary of studies on the safety of quercetin supplement.

*n*	Health Status	Dosage (mg)	Duration (weeks)	Adverse Event	Ref.
93	Normotensive and Stage I hypertension	150	6	No effects on liver, kidneys, electrolytes, and hematology biomarkers	[73]
49	Healthy	150	8	Increased TNF-α by 0.11 pg/*mL*, p < 0.05	[89]
40	Athletes	1000	6	No adverse events reported	[90]
30	Chronic Hepatitis C	250–5000	4	No adverse effects on liver function but mild gastrointestinal discomfort	[91]

Abbreviations: HTN, hypertension; TNF-α, tumor necrosis factor.
